# Two cases of hard metal lung disease showing gradual improvement in pulmonary function after avoiding dust exposure

**DOI:** 10.1186/s12995-015-0070-9

**Published:** 2015-08-04

**Authors:** Hiroya Terui, Satoshi Konno, Kichizo Kaga, Yoshihiro Matsuno, Kanako C. Hatanaka, Hiromi Kanno, Hiroshi Moriyama, Motohiro Uo, Masaharu Nishimura

**Affiliations:** First Department of Medicine, Hokkaido University School of Medicine, Kita-15 Nishi-7 Kita-Ku, Sapporo, Hokkaido 060-8638 Japan; Cardiovascular and Thoracic Surgery, Hokkaido University School of Medicine, Sapporo, Hokkaido 060-8638 Japan; Department of Surgical Pathology, Hokkaido University Hospital, Sapporo, Hokkaido 060-8638 Japan; Niigata University Medical & Dental Hospital Bioscience Medical Research Center, Niigata, Niigata 951-8520 Japan; Advanced Biomaterials, Department of Restorative Sciences, Division of Oral Health Sciences, Graduate School of Medical and Dental Sciences, Tokyo Medical and Dental University, Bunkyo-ku, Tokyo 113-8549 Japan

**Keywords:** Hard metal lung disease, Pulmonary function, Dust exposure, Avoidance, BALF

## Abstract

We present herein two cases of hard metal lung disease (HMLD) with distinct pathological findings. Both cases showed gradual improvements in pulmonary function over a period of a few years (Case 1: 30 months; Case 2: 12 months) after the avoidance of dust exposure, while improvements on high-resolution computed tomography were modest. The increased lymphocytes and decreased CD4/CD8 ratio in BALF observed at initial diagnosis normalized after the avoidance of dust exposure in one case. To the best of our knowledge, this is the first report demonstrating continual follow-up of pulmonary function and radiographic findings, and a comparison of BALF findings before and after avoidance of hard metal dust exposure.

## Background

Hard metal lung disease (HMLD) is a rare occupational lung disease that can occur in workers engaged in the manufacture, utilization, or maintenance of tools composed of hard metal, a synthetic compound composed of tungsten carbide and cobalt [1, 2]. Although giant cell interstitial pneumonitis (GIP) is known as a typical pathological finding for this disease [3–5], some cases without GIP have also been reported [6, 7]. The clinical course of this disease varies, with some cases requiring corticosteroid treatment and others showing spontaneous improvement simply by avoiding exposure to hard metal [5, 8–12]. We report two cases of HMLD with distinct pathological findings: one as typical GIP; and the other characterized by peribronchial fibrosis without typical features of GIP. Continual follow-up of pulmonary function and radiological findings as well as comparisons of bronchoalveolar lavage fluid (BALF) findings before and after dust exposure were obtained.

## Case presentation

### Case 1

**Fig. 1:**
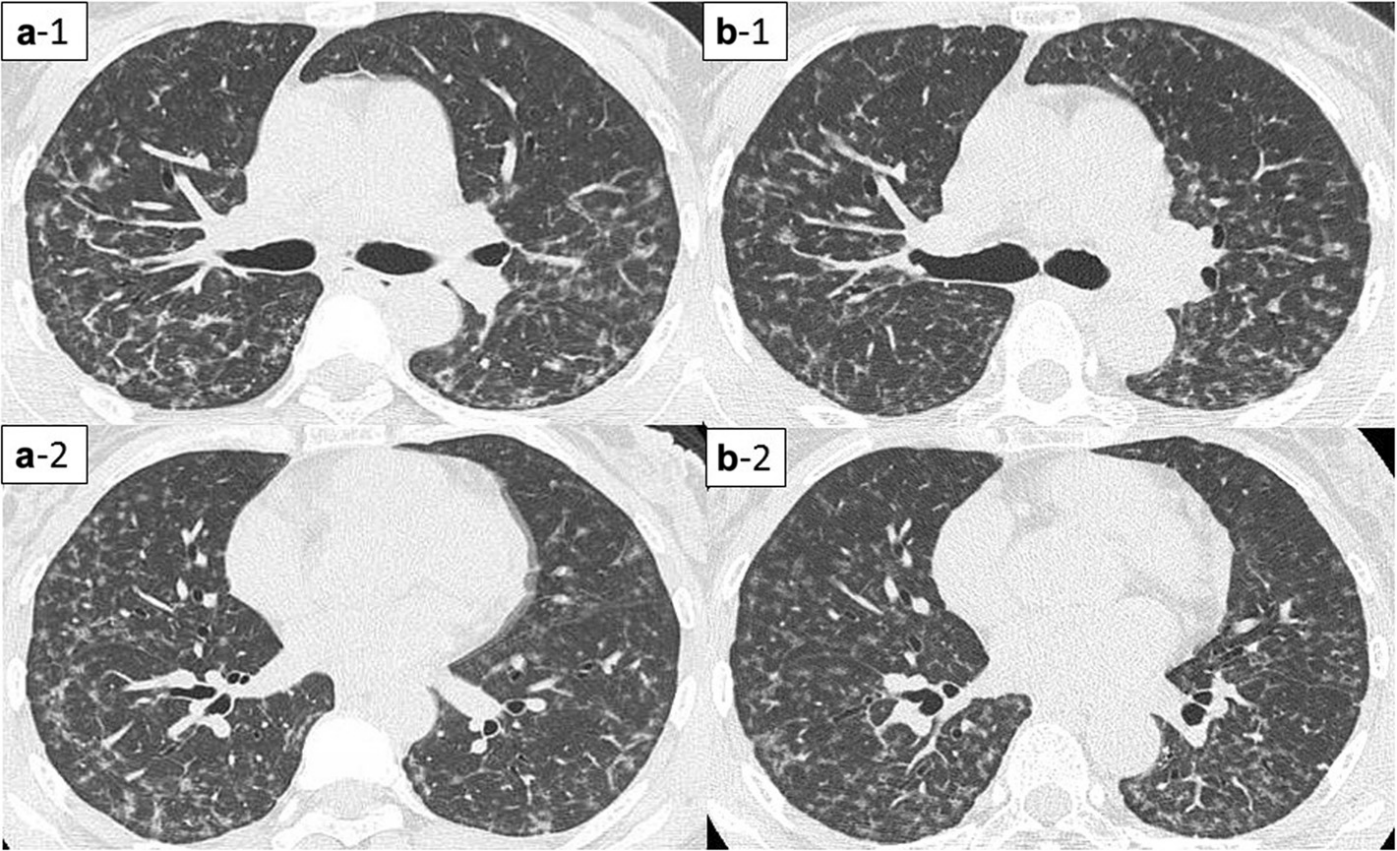
High-resolution computed tomography (HRCT) findings before and after the avoidance of dust exposure in Case1. Bilateral diffuse centrilobular micronodular opacities and curved linear shadows in the dorsum. **a**: before the avoidance (a-1: upper lobe; a-2: lower lobe); **b**: after the avoidance (b-1: upper lobe; b-2: lower lobe)

**Fig. 2:**
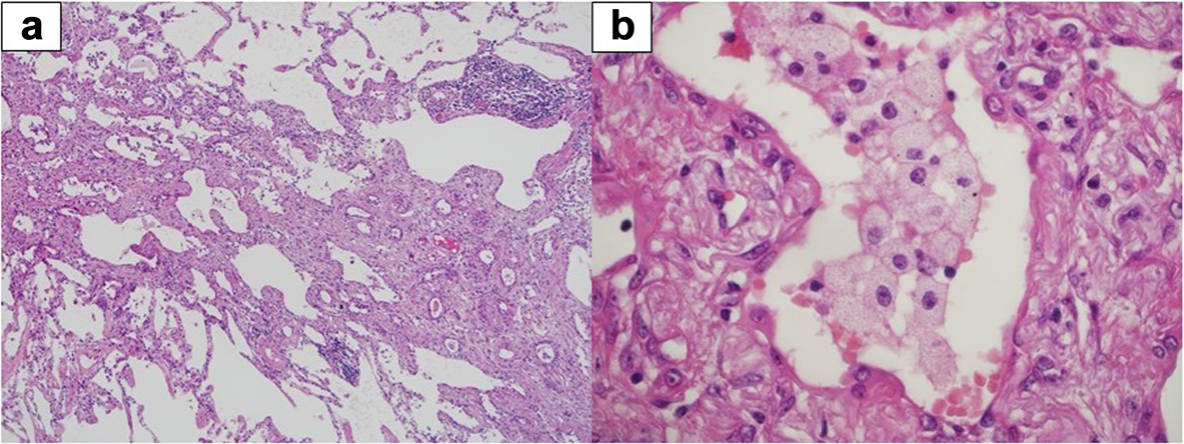
Histological findings from biopsy specimens in Case 1. Peribronchiolar fibrosis and lymphocytic infiltration are marked, but multinucleated giant cells are barely apparent (**a**: hematoxylin and eosin [HE] stain × 100; **b**: HE × 400)

**Fig. 3:**
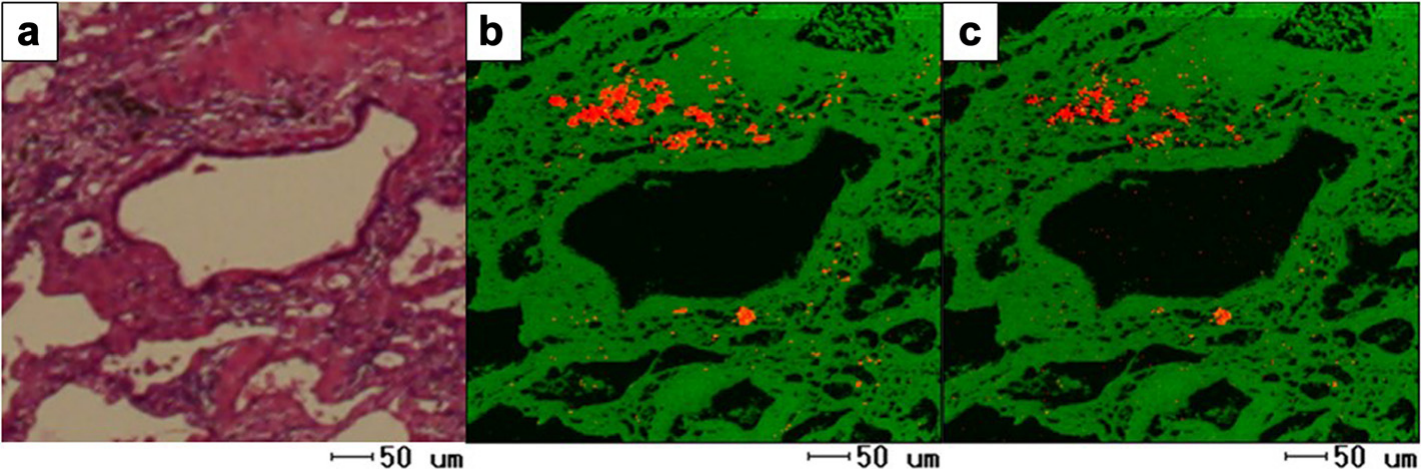
Images of EPMA in Case 1. The presence of tungsten and cobalt in the peribronchial fibrotic region was observed (**a**: HE stain; **b**: tungsten; **c**: cobalt)

A 50-year-old non-smoking woman presented with a 6-month history of nonproductive cough, which had started 8 years after she began working with a hard metal grinder without using a dust protective mask. High-resolution computed tomography (HRCT) of the chest showed diffuse centrilobular micronodular opacities and curved linear shadows in the dorsum (Fig. 1a). Pulmonary function test results were almost within normal levels for vital capacity (VC) (2.20 L; percentage predicted VC (%VC), 86.6 %), forced expiratory volume in 1 s (FEV_1_) (2.07 L; percentage predicted FEV_1_ (%FEV_1_), 95.4 %), total lung capacity (TLC) (3.33 L; percent predicted TLC (%TLC), 87.2 %), and diffusing capacity of the lung for carbon monoxide (%DL_CO_; 77.8 %). BALF obtained from the right middle lobe (S^5^) showed an increased percentage of lymphocytes (55.0 %) and a decreased CD4/CD8 ratio (0.40) (Table 1). Multinucleated giant cells were not observed in BALF. Thoracoscopic lung biopsy (video-assisted thoracoscopic surgery (VATS)) of the right upper and lower lobes was performed, and microscopic examination revealed peribronchiolar fibrosis with lymphocytic infiltration. Although a few multinucleated giant cells were observed, no features diagnostic of GIP were evident (Fig. 2). Elemental analysis of the lung tissue using an electron probe microanalyzer with a wavelength-dispersive spectrometer (EPMA-1610; Shimadzu Ltd, Kyoto, Japan) revealed the presence of tungsten and cobalt in the peribronchial fibrotic region (Fig. 3). Furthermore, X-ray absorption fine structure (XAFS) analysis confirmed that the tungsten present in the samples was in the form of tungsten carbide (data not shown). These results clearly indicated that the subject had inhaled hard metal, leading to the diagnosis of HMLD.

**Table 1:** Results from bronchoalveolar lavage fluid (BALF) in Case 1

	Before avoidance of dust exposure	After avoidance of dust exposure
Lavaged area	rt. S^5^	rt. S^5^
Total cell count (/ml)	3.36 × 10^5^	4.04 × 10^5^
Macrophages (%)	43.8	72.6
Lymphocytes (%)	55.0	26.3
Neutrophils (%)	0.6	1.0
Eosinophils (%)	0.0	0.2
CD4/CD8 ratio	0.40	1.72
Multinucleated giant cells	(-)	(-)

**Fig. 4:**
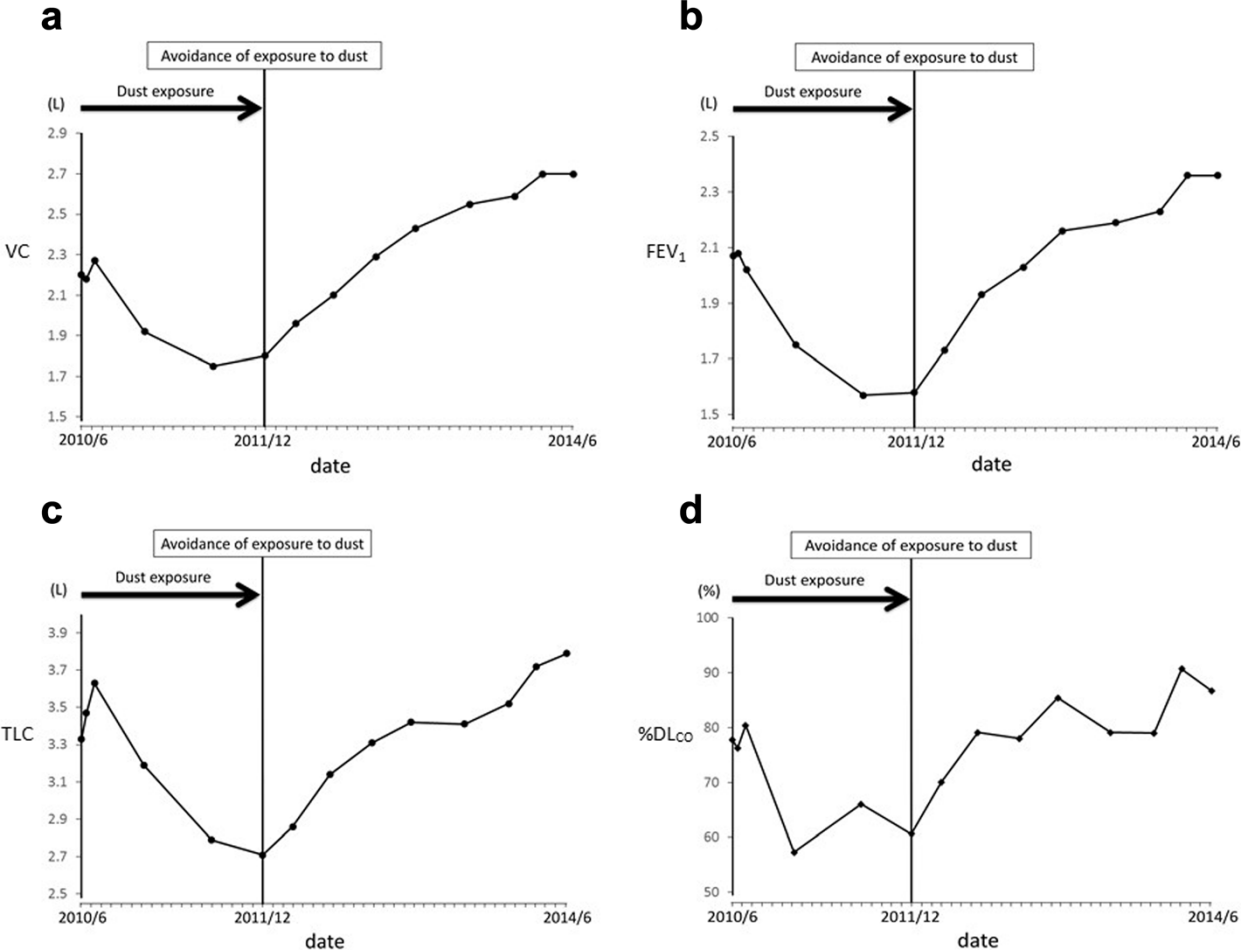
Serial changes in pulmonary function in Case 1. **a**: vital capacity (VC), **b**: forced expiratory volume in 1 s (FEV1), **c**: total lung capacity (TLC), **d**: diffusing capacity of the lung for carbon monoxide (%DL_CO_)

After reaching this diagnosis, we recommended the patient quit her job or transfer to a different workplace to completely avoid exposure to hard metal dust. However, she continued working at the same place without using a dust protective mask for financial reasons, and nonproductive cough continued. In addition, pulmonary function continued to worsen. Finally, 18 months after diagnosis, she obtained a transfer to a different department where she was completely isolated from exposure to hard metal dust. Her cough improved and had completely disappeared by 2 months after ending exposure to hard metal dust. A series of pulmonary function tests revealed continuous improvements over the course of 30 months without exposure to hard metal dust (Fig. 4), although improvements in HRCT findings were modest (Fig. 1b). BALF findings obtained at 21 months after ceasing hard metal dust exposure showed a decreased percentage of lymphocytes (26.3 %) and an increase in the CD4/CD8 ratio (1.72) compared to the initial findings (Table 1). She did not take any corticosteroids or other immunosuppressive drugs during this follow-up period.

### Case 2

**Fig. 5:**
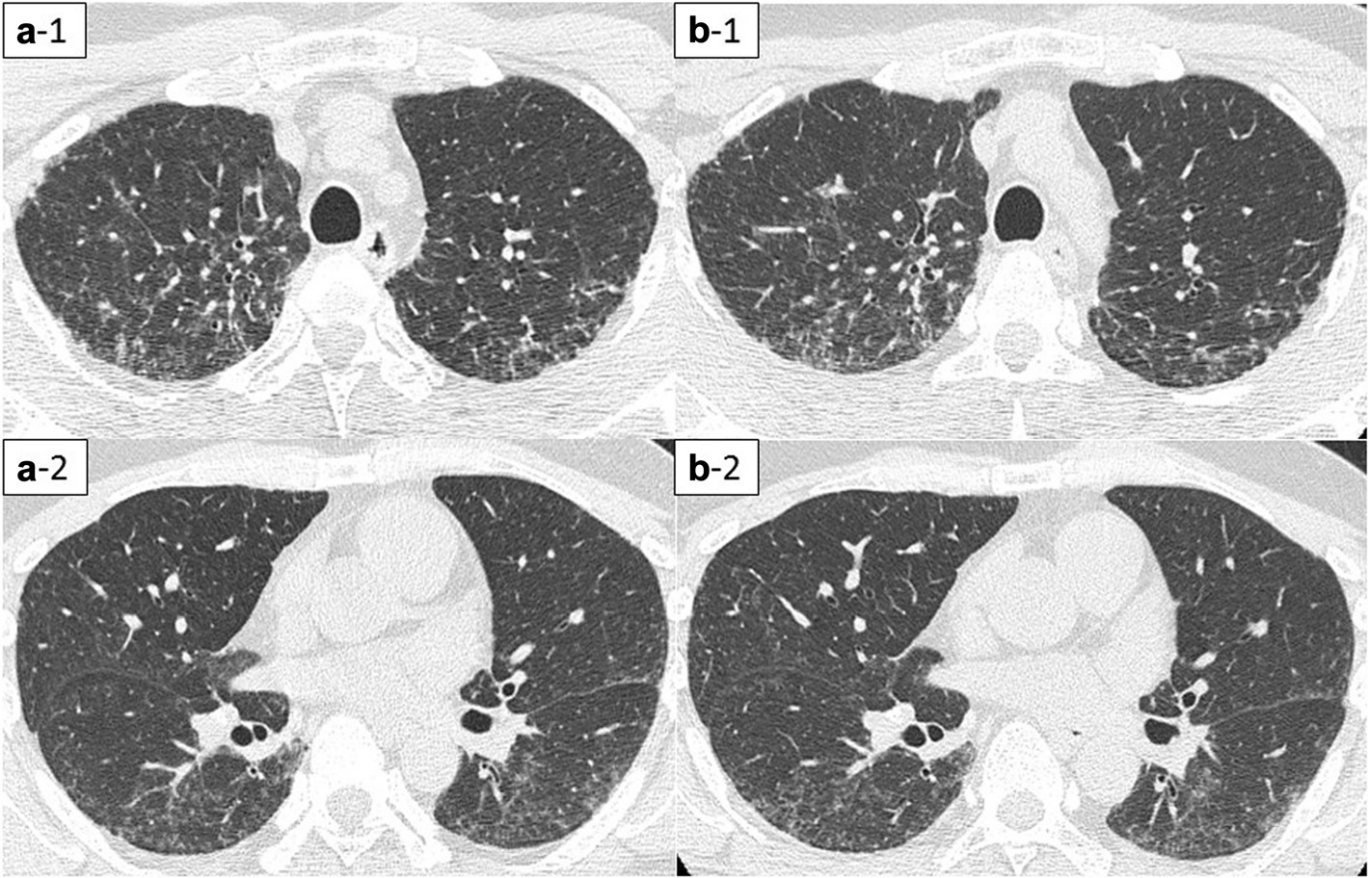
HRCT findings before and after the avoidance of exposure in Case 2. Bilateral diffuse centrilobular micronodular opacities and reticular shadows. **a**: before the avoidance (a-1: upper lobe; a-2: lower lobe); **b**: after the avoidance (b-1: upper lobe; b-2: lower lobe)

**Fig. 6:**
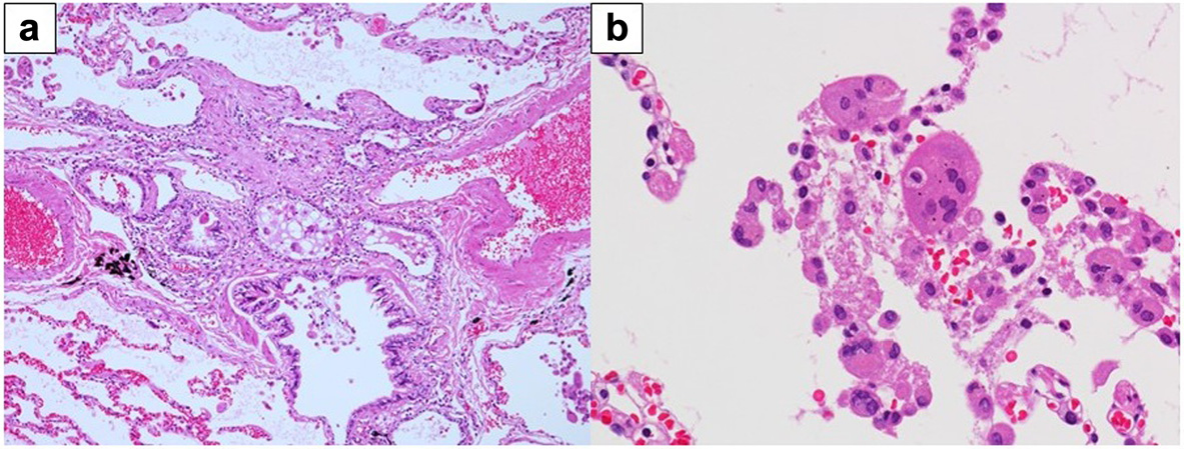
Histological findings from biopsy specimens in Case 2. Marked peribronchiolar fibrosis accompanies multinucleated giant cells in the bronchioles and alveoli (**a**: HE stain × 100; **b**: HE stain × 400)

**Fig. 7:**
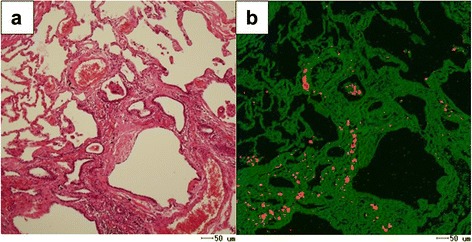
Images of EPMA in Case 2. The presence of tungsten in the bronchioles and alveoli was observed (**a**: HE stain; **b**: tungsten)

A 45-year-old non-smoking man presented with a 2-year history of dyspnea, which had started 11 years after he began a job mixing raw materials for manufacturing hard metal tools without using a dust protective mask. Chest HRCT showed diffuse centrilobular micronodular and reticular shadows (Fig. 5a). Pulmonary function testing showed a low VC (2.10 L; %VC, 46.0 %), FEV_1_ (1.61 L; %FEV_1_, 41.9 %), TLC (3.32 L; %TLC, 55.9 %), and DL_CO_ (%DL_CO_, 65.0 %). Although BALF obtained from the right middle lobe (S^5^) demonstrated normal cell counts, multinucleated giant cells were observed (Table 2). VATS of the left upper and lower lobes were performed, and histopathological examination revealed interstitial fibrosis accompanied by multinucleated giant cell infiltration into peribronchiolar and alveolar tissues, leading to a diagnosis of GIP (Fig. 6). Elemental analysis of the lung tissue revealed the presence of tungsten in the bronchioles and alveoli (Fig. 7), and XAFS analysis confirmed the presence of tungsten carbide (data not shown), thus leading to the clinical diagnosis of HMLD.

**Table 2:** Results from bronchoalveolar lavage fluid (BALF) in Case 2

	Before avoidance of dust exposure	After avoidance of dust exposure
Lavaged area	rt. S^5^	rt. S^5^
Total cell count (/ml)	1.66 × 10^5^	6.40 × 10^4^
Macrophages (%)	86.9	84.4
Lymphocytes (%)	10.7	13.8
Neutrophils (%)	2.1	0.6
Eosinophils (%)	0.3	1.2
CD4/CD8 ratio	1.85	1.22
Multinucleated giant cells	(+)	(+)

**Fig. 8:**
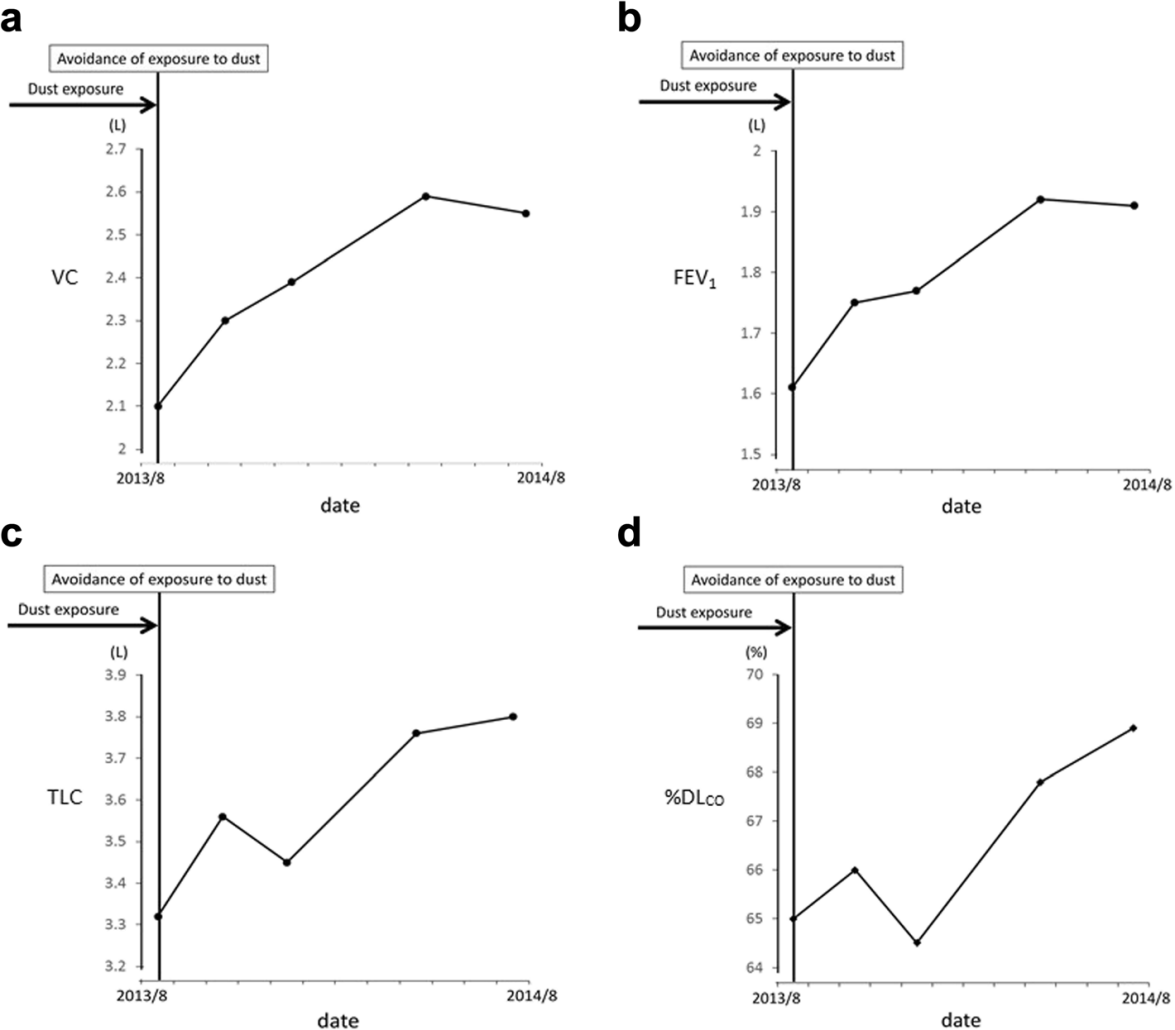
Serial changes in pulmonary function in Case 2. **a**: VC; **b**: FEV_1_; **c**: TLC; **d**: %DL_CO_

Three weeks after his first visit, the patient obtained a transfer to a different department with reduced (but not eliminated) exposure to dust. Dyspnea gradually improved after reducing the dust exposure. Although a series of pulmonary function tests revealed improvements over the course of a year (Fig. 8), improvements in HRCT findings were modest (Fig. 5b). Multinucleated giant cells remained observable in BALF a year after reducing dust exposure, and cell differentiation was similar to that before the avoidance of dust exposure (Table 2). The patient did not take any corticosteroids or other immunosuppressive drugs during this follow-up period.

## Discussion

The clinical features of HMLD are known to be heterogeneous in several respects, such as pathological findings, BALF findings, and clinical course. In the two cases we encountered, thoracoscopic lung biopsy revealed distinct pathological findings, with one case characterized by typical GIP, and the other by diffuse centrilobular fibrosis around the respiratory bronchioles without GIP. In a study of 100 cases of HMLD, Asghar et al. [6] described 41 cases not showing GIP findings, and some cases only showed infiltration of lymphocytes and fibrosis in peribronchial lesions similar to that noted in our Case 1. Tanaka et al. [7] recently examined 19 cases of HMLD diagnosed in Japan and described various pathological findings, including typical GIP, centrilobular fibrosis, and usual interstitial pneumonia (UIP) pattern. In the present cases, differences in the type of job (Case 1: grinder of hard metal; Case 2: mixer of raw materials) and/or genetic backgrounds of the two patients may have been involved in the different pathological findings observed.

Avoidance of exposure to hard metal dust is central to preventing deterioration of this disease in any case, and improvements of the symptoms and/or radiological findings after avoiding exposure have been reported in several previous studies [5, 8–10]. However, to the best of our knowledge, no previous reports have provided continuous follow-up information on pulmonary function and radiological findings after reducing exposure to hard metal dust exposure. Of note, pulmonary function displayed gradual improvement after reducing dust exposure in both cases, while improvements in HRCT findings were more modest. As shown in Case 1, the improvement in pulmonary function was continuing even 2 years after starting avoidance of hard metal dust. Clinicians should thus keep in mind that gradual improvement of pulmonary function can be expected over the course of years, despite the lowered pulmonary function before the avoidance of dust exposure. Immunological responses to hard metal ingredients trapped in the bronchioles and/or to cobalt dissolved in the lungs might gradually diminish after marked reductions in exposure. We therefore speculate that the persistence of hard metal in the lung may determine the clinical course of the disease after the avoidance of dust exposure.

In some previously reported cases, respiratory failure progressed to death despite suspending exposure to the dust [11, 12], while in others, the symptoms and/or radiological findings improved with the avoidance of exposure [8–10]. The pathogenesis of HMLD is thus considered to encompass a wide spectrum of cases, from mimicking hypersensitivity pneumonitis to those similar to typical pneumoconiosis generally caused by silica and asbestos, progressing even after the avoidance of dust exposure.

Although previous studies have suggested that increased lymphocytes and decreased CD4/CD8 ratio are characteristic BALF findings in subjects with HMLD [7, 9, 12, 13], this does not always seem to be the case. In fact, in the present study, BALF findings from Case 2 showed normal cell differentiation and CD4/CD8 ratio. To date, no reports have compared BALF findings of subjects with HMLD before and after the avoidance of dust exposure similar to that encountered in the present study. The increased lymphocytes and decreased CD4/CD8 ratio observed at initial diagnosis in our Case 1 normalized by 9 months after reducing dust exposure, indicating altered immune cellular responses to hard metal in the pathogenesis of this case.

## Conclusion

We have presented two cases of HMLD with continuous follow-up of pulmonary function and radiological findings before and after avoidance of hard metal dust exposure. The clinical courses of the two cases were presented, together with a comparison of BALF findings before and after avoidance of dust exposures.

## Consent

Written informed consent was obtained from both patients for the publication of this case report and the accompanying images.
